# Adaptive visual–tactile fusion recognition for robotic operation of multi-material system

**DOI:** 10.3389/fnbot.2023.1181383

**Published:** 2023-06-20

**Authors:** Zihao Ding, Guodong Chen, Zhenhua Wang, Lining Sun

**Affiliations:** The Jiangsu Provincial Key Laboratory of Advanced Robotics, Soochow University, Suzhou, China

**Keywords:** tactile image, robotic recognition, visual-tactile fusion, dropout, VTN model

## Abstract

The use of robots in various industries is evolving from mechanization to intelligence and precision. These systems often comprise parts made of different materials and thus require accurate and comprehensive target identification. While humans perceive the world through a highly diverse perceptual system and can rapidly identify deformable objects through vision and touch to prevent slipping or excessive deformation during grasping, robot recognition technology mainly relies on visual sensors, which lack critical information such as object material, leading to incomplete cognition. Therefore, multimodal information fusion is believed to be key to the development of robot recognition. Firstly, a method of converting tactile sequences to images is proposed to deal with the obstacles of information exchange between different modalities for vision and touch, which overcomes the problems of the noise and instability of tactile data. Subsequently, a visual-tactile fusion network framework based on an adaptive dropout algorithm is constructed, together with an optimal joint mechanism between visual information and tactile information established, to solve the problem of mutual exclusion or unbalanced fusion in traditional fusion methods. Finally, experiments show that the proposed method effectively improves robot recognition ability, and the classification accuracy is as high as 99.3%.

## 1. Introduction

The growing individualization of products demands facilities that can manufacture small batch sizes with little effort. Autonomous robots can help increase the required flexibility. The reliable task execution is required for similar but different product variants. The Institute of Robotics and Mechatronics of the German Aerospace Center (DLR) has developed an autonomous robot assembly system for flexible manufacturing (Nottensteiner et al., 2021). Take the gyroscope as an example, it is an instrument that can accurately determine the orientation of moving objects, which is of great strategic importance to industry, defense, and other high-tech development. For the high-precision gyroscope gluing process, it is necessary to consider the bonding strength between the parts and the reliability of the bonding seal (Yan et al., 2023). The bonding strength is related to the material properties, and there is a significant difference between metal and non-metal parts. The gyroscope is a combination of metal parts and non-metal parts. We call this a multi-material system, which requires us to correctly identify the material properties of different parts when assembling. The vision sensor can only acquire the appearance characteristics of the object but cannot perceive the material properties of the object. Moreover, vision is susceptible to the influence of light and background, and the recognition rate is not high in industrial scenes (Haibo et al., 2023). Therefore, we need to combine vision and touch together to recognize objects.

The integration of visual and tactile information is challenging, and there is no standard framework for doing this work. In addition, the difficulty with integrating tactile force feedback without leading to potentially disrupting oscillations in the robotic system. Additionally, humans can process and integrate a large amount of sensory information simultaneously. Thus, it is also expected that robots can perceive the attributes of objects as accurately as humans (Dunin-Barkowski and Gorban, 2023). The object recognition algorithm based on visual images has been developed in many fields (De Vries et al., 2019; Qi et al., 2021; Grimaldi et al., 2022). However, the scenes with similar objects and object occlusion pose significant challenges to the visual algorithm. In this regard, the tactile sensor can be installed on the dexterous hand (Sundaralingam et al., 2019; Wang et al., 2019, 2022) to sense the material attributes of the object, such as texture (Tsuji and Kohama, 2019; Park et al., 2021) and roughness (Işleyen et al., 2019), to make up for the shortcomings of the visual sensor.

Although great progress has been made in object recognition using visual or tactile information alone, the attributes of objects are diverse and a single sensor alone cannot fully recognize objects. Shah et al. (2021) reviewed the design and development of the vision-based tactile sensors (VBTSs). Yuan et al. (2017) compared the recognition performance on a single modality when joint trained on one or two modalities and found that the extra information from another modality would boost the performance on a single-modality match. Li et al. (2019) adopted a generative adversarial learning-based prediction model and proved that visual modality and tactile modality could be converted to each other. As the two perceptual modalities can provide complementary information, visual-tactile fusion learning contributes to robot target recognition (Wang et al., 2018). How to fuse the information from the two different modalities is challenging (Gao et al., 2022). Sun et al. (2016) proposed a systematic system for target identification and manipulation via visual and tactile data. Liu et al. (2016, 2018a,b) proposed a joint group kernel sparse coding (JGKSC) method to tackle the intrinsically weak pairing problem in visual-tactile data samples and subsequently developed a visual cross-modal matching algorithm designed a shared dictionary learning model. Yang et al. (2017) and Xiong et al. (2021) used the above coding approach to recognize visually similar objects. Zheng et al. (2020) addressed visual-tactile cross-modal learning in the lifelong learning setting by establishing a knowledge-forgetting mechanism. The above studies validated the effectiveness of the proposed visual-tactile cross-modal matching framework and method through extensive experiments.

The deep learning techniques (LeCun et al., 2015) provide additional fusion models for visual-tactile fusion. He et al. (2022) first reviewed the physiological basis of biological vision and tactile systems and the biological vision-tactile fusion mechanism. Zheng et al. (2016) presented a novel deep learning method dealing with the surface material classification problem based on a Fully Convolutional Network (FCN). Gao et al. (2016) established a deep learning model that could simultaneously input visual data and tactile data for training, and the experiment showed that the fusion effect was superior to the model that only uses visual data or tactile data for training. Calandra et al. (2018) used deep reinforcement learning and combined data from tactile sensors and image acquisition as network inputs to grasp objects, improving their success rate in grasping experiments. Cui et al. (2020) proposed a novel 3D convolution-based visual-tactile fusion deep neural network (C3D-VTFN) to evaluate the grasp state. Lee et al. (2019) built a multi-modal representation learning model to effectively use tactile and visual feedback for hole search. Zhang et al. (2020, 2021a,b) proposed a framework for object clustering based on visual-tactile fusion and graph learning. Chaudhury et al. (2022) fused vision and touch for pose estimation and found that tactile imaging was used to further refine contact point and object pose estimation. Shi et al. (2023) designed a visual-tactile fusion road recognition system for autonomous vehicles. Babadian et al. (2023) proposed four efficient models based on dynamic neural network architectures for unimodal and multimodal object recognition.

**Figure 1:**
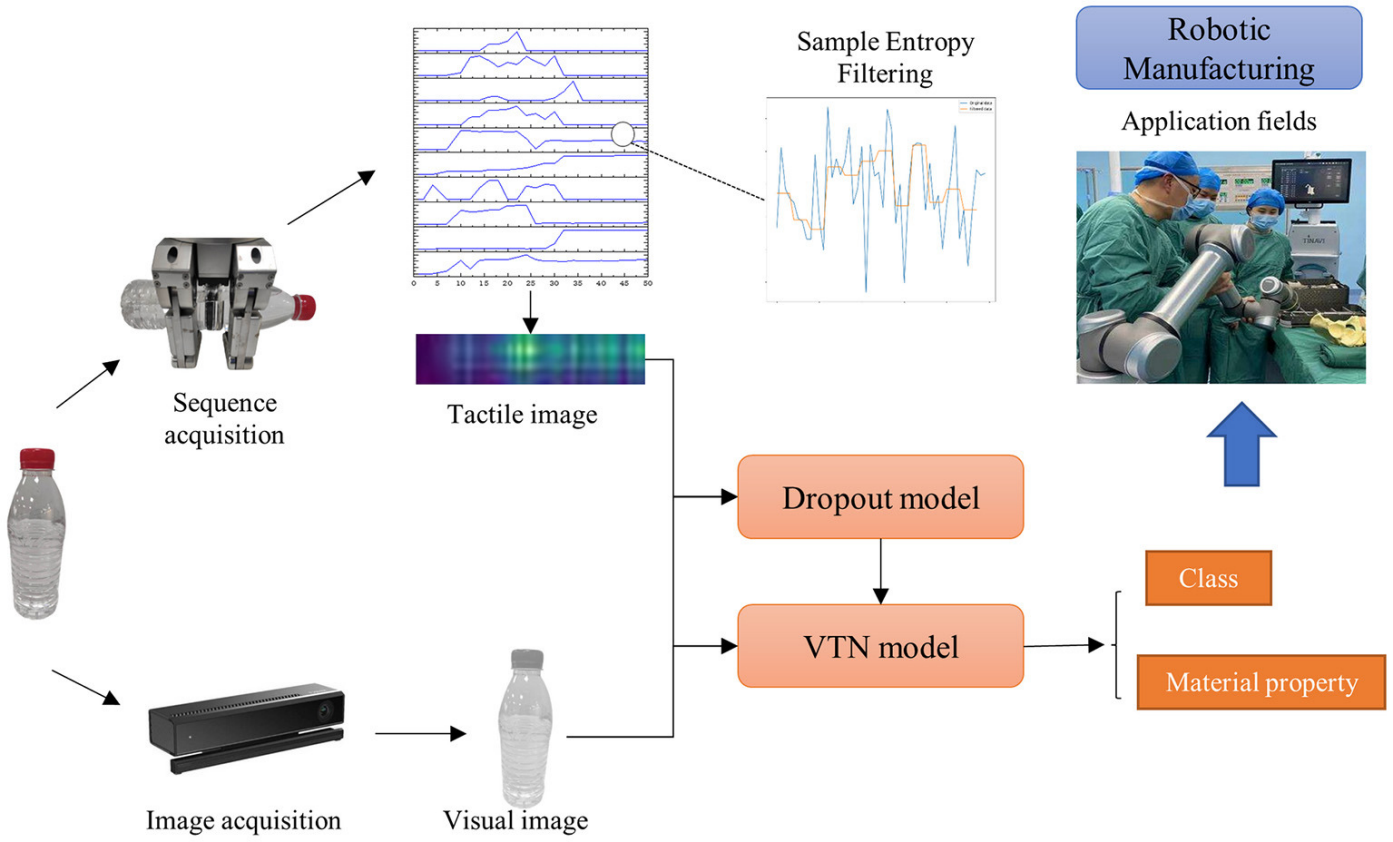
The process of object identification in this paper. The proposed VTN model outputs the class and material information by fusing visual-tactile information to help robots perform more elaborate manipulation tasks.

The research on the fusion of visual and tactile features is still in its early stages, and there is still much room for improvement in the design of visual and tactile feature fusion (Zhang et al., 2020; Ruan et al., 2023). The modal differences between vision and touch remain the biggest obstacle to the direct application of these methods, such as the big differences in receiving fields and signal types and the frequency of visual and tactile data. The existing methods often process the visual and tactile information separately and fuse them at the decision-making level, which will weaken the intrinsic connection between them. Moreover, these methods usually assume that each sample's visual and tactile data are available. However, there are incomplete scenes, missing visual or tactile data, or the role of visual and tactile information in the recognition results is not analyzed by some methods even if the information is complete. In addition, in tactile data acquisition, the robot collects tactile data through continuous contact, which is easily disturbed or interrupted by the external environment due to noise. However, the existing methods do not filter the tactile data, and there is no practical way to deal with the influence of noise and information loss. Based on the development of existing research algorithms, we explore a new framework and put forward improvements, as shown in Figure 1. Firstly, a method of converting tactile sequences to images is proposed to deal with the obstacles of information exchange between different modalities for vision and touch. Then, with visual images and tactile images as input, the quality assessment network and target recognition network are trained, respectively. The output of the quality assessment network is used as the dropout parameter of the network fusion layer to adjust the proportion of the two kinds of information in the recognition to improve the performance of the network.

Our contribution can be summarized into the following three parts.

1. First, tactile time sequences are encoded into images so that tactile and visual information are unified into the same modality. And a tactile image filtering algorithm based on the sample entropy is proposed to improve the reliability of tactile data.
2. A visual-tactile fusion framework is constructed, which takes both visual and tactile data as input, integrates the advantages of both, and has a simple structure.
3. The adaptive dropout algorithm is added to the fusion framework, which can adjust the neuron connection strength of the fusion layer network according to the quality of the visual and tactile samples, and control the effect of the two in the model, greatly improving the recognition effect and anti-interference ability of the model.

## 2. 2D tactile image based on the sample entropy filtering

The development of neural networks in image recognition has made great strides, whereas progress has been slow in time sequence recognition scenes. This is due to the uncertainty regarding the length of time sequences, along with the indistinct characteristics that follow after visualization. As a result, the current recognition algorithm for time sequence data remains immature, with no unified framework for processing them. In this section, we propose a model that can transform one-dimensional data into two-dimensional images, as well as introduce the process of image denoising based on sample entropy. The model transformation lays the foundation for subsequent information fusion.

### 2.1. 2D tactile image model

Our tactile data consists of a 1D time sequence that is composed of feedback force continuously collected by the sensor, as demonstrated in Formula (1). Here, n refers to the number of tactile sensors, while *F* refers to the force received by the sensors.


(1) $$\begin{array}{l} F=\left[F_{1},\;F_{2},\ldots ,F_{n}\right] \end{array}$$

Representative time sequence recognition networks such as RNN and LSTM mainly focus on natural language processing and speech recognition, but there is no common 1D recognition network framework for object recognition. Therefore, we propose a method to convert the 1D tactile sequences from the tactile sensor array on a multi-fingered dexterous hand into a 2D image model.

An image is typically represented as a matrix in a computer, with the pixel being the most basic unit for digital image processing. Therefore, the elements that constitute an image are its size and pixel values. By considering the number of sensors in the tactile sensor array as the image's width, the number of consecutive acquisitions as its height, and the feedback value collected from each sensor as the pixel value, the tactile image can be expressed using Formula (2).


(2) $$\begin{array}{l} I=\left(\begin{array}{c} F_{11}\;\cdots \;F_{1n}\\ \vdots \;\ddots \;\vdots \\ F_{m1}\;\cdots \;F_{mn} \end{array}\right) \end{array}$$

**Figure 2:**
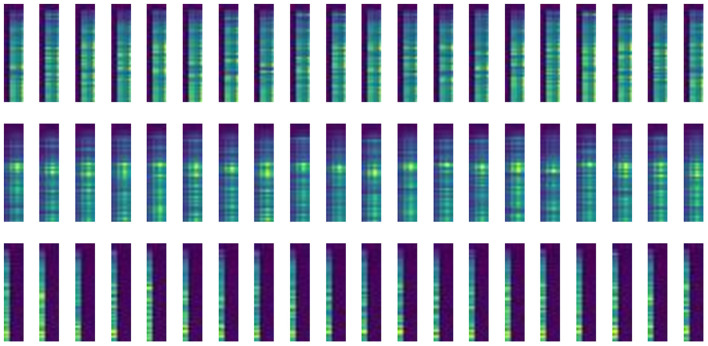
Example of the tactile images. Tactile images are acquired by a tactile sensor array attached to a multi-fingered hand and have undergone image conversion. The image width of 10 represents the number of tactile sensors, and the image height of 50 represents the length of the acquired tactile sequence.

Where, m refers to the acquisition frequency, while n refers to the number of tactile sensors. The tactile sensor array we have built consists of 10 tactile sensors, which means the width of the tactile image is ten. The acquisition frequency of the tactile sensor is 20 ms. Taking 50 consecutive groups of tactile sensor data and treating them as a group, the image height is set to 50. The processed image is visualized as shown in Figure 2.

### 2.2. Sample entropy filtering

We have collected pressure feedback values from the tactile sensors on the fingers while grasping an object with the dexterous hand. For a given object, the variation of tactile data should ideally lie within a specific range. However, external factors such as unstable finger output force and object sliding can introduce noise into the tactile data. To mitigate the effects of such noise on the data, it is necessary to apply a filtering technique.

**Figure 3:**
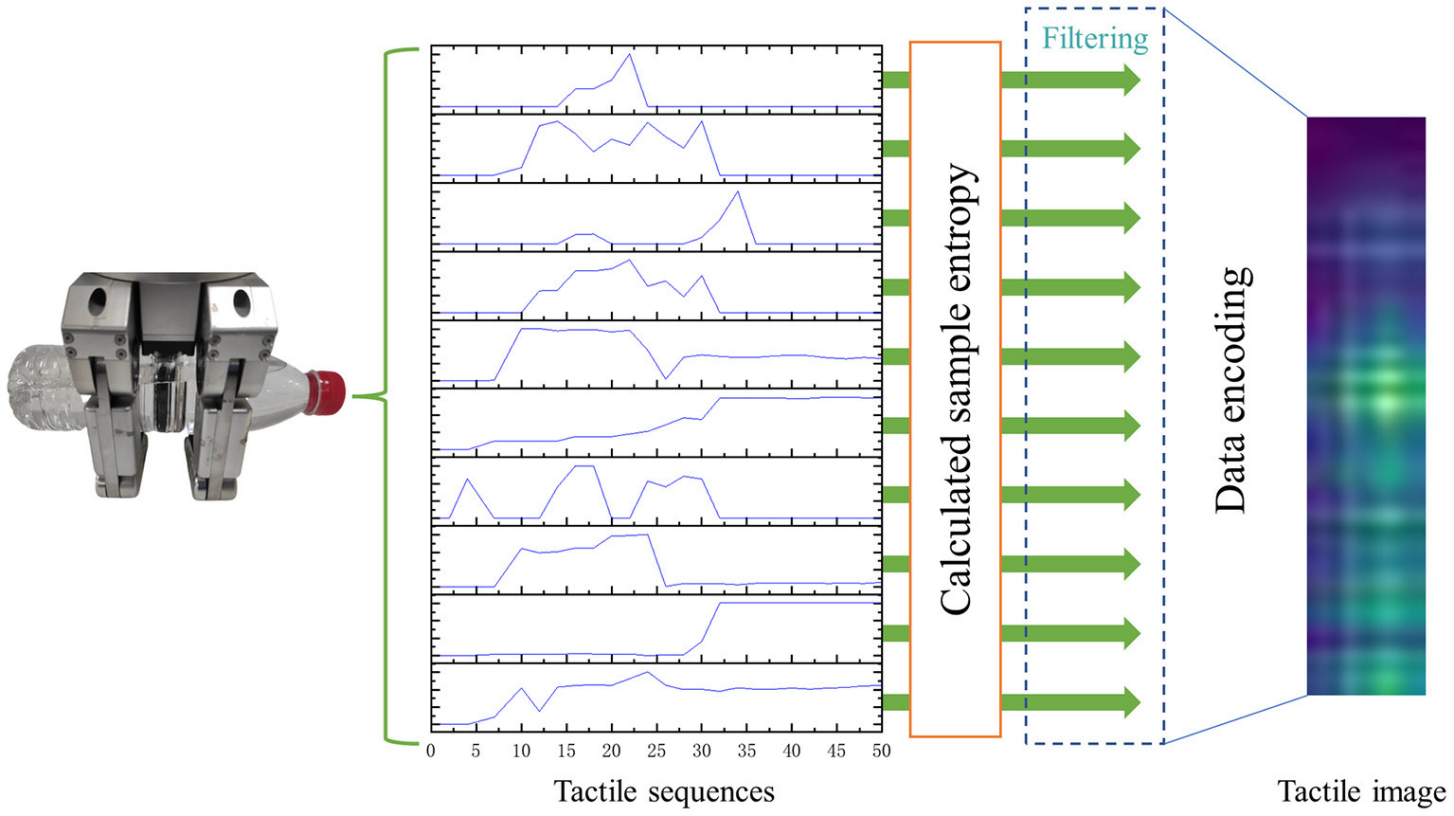
The process of the adaptive median filter. The adaptive median filter changes the filtering window size according to the calculated sample entropy to denoise the tactile data.

While conventional median filters use a fixed window size, they are often unable to balance between preserving image details and denoising efficiently. To address this limitation, we propose an adaptive median filter that utilizes the sample entropy factor, as illustrated in Figure 3. During the filtering process, the adaptive median filter adjusts the filtering window size according to the calculated sample entropy.

The sample entropy is a method for measuring the complexity of time sequences first proposed by Richman et al. (2004). It can evaluate the repeatability of the waveform, that is, the frequency. The larger the entropy, the higher frequencies in the waveform and the more the noise (Delgado-Bonal and Marshak, 2019). To address the issue of noise within tactile images, we propose an image filtering method based on the sample entropy method, which operates as follows.

The image matrix is divided into columns to obtain n column vectors, as shown in formula (3).


(3) $$\begin{array}{l} M=\left[S_{0},\;S_{1},\ldots ,S_{n-1}\right] \end{array}$$

Each column vector corresponds to the time sequences collected by a single sensor, as shown in formula (4).


(4) $$\begin{array}{l} S_{i}={\left[F_{1i},\;F_{2i},\ldots ,F_{mi}\right]}^{T},\;i=0\sim n-1 \end{array}$$

Next, the sample entropy is calculated for each column vector. For any column vector, a threshold r representing the similarity comparison is specified, and a metric m for dividing the length of the subsequences is determined. (N–m+1) subsequences can be obtained by reconstructing the original sequences, as shown in (5).


(5) $$\begin{array}{l} S_{i}^{m}=\left[S_{i}^{m}\left(1\right),\;S_{i}^{m}\left(2\right),\ldots ,S_{i}^{m}\left(N-m+1\right)\right] \end{array}$$

Each subsequence is represented by $S_{i}^{m}\left(u\right)$, as shown in formula (6).


(6) $$\begin{array}{l} S_{i}^{m}\left(u\right)={\left[F_{ui},\;F_{\left(u+1\right)i},\ldots ,F_{\left(u+m-1\right)i}\right]}^{T} \end{array}$$

The distance D_uv_ between any two reconstructed vectors $S_{i}^{m}\left(u\right)$ and $S_{i}^{m}\left(v\right)$ is calculated, where/is the maximum value in the absolute value of the difference between the corresponding elements of the two vectors. The formula is shown in (7).


(7) $$\begin{array}{l} D_{uv}=D\left[S_{i}^{m}\left(u\right),\;S_{i}^{m}\left(v\right)\right]=\max\left[\left|\overset{i}{\underset{u+w}{F}}-F_{v+w}^{i}\right|\right],\\ w=0\sim m-1 \end{array}$$

Then, formula (8) is used to count the number of vectors that meet the following conditions.


(8) $$\begin{array}{l} N_{d<r}=count\left[D_{uv}<r\right],\;u=0\sim N-m+1,\\ v=0\sim N-m+1,\;u\neq v \end{array}$$

Formula (9) is used to calculate the ratio (the number of satisfied conditions to the total number), representing the similarity between the subsequences and the original sequences.


(9) $$\begin{array}{l} SD_{m}^{r}=\frac{N_{d<r}}{\left(N-m+1\right)\left(N-m\right)} \end{array}$$

The similarity at m+1 is calculated according to steps (5–9) above, and the sample entropy is calculated according to formula (10).


(10) $$\begin{array}{l} En\left(S_{i}\right)=-\ln\;\left[\frac{SD_{m+1}^{r}}{SD_{m}^{r}}\right] \end{array}$$

Repeating steps (5–10) above to calculate the sample entropy of the time sequences for all column vectors, we finally obtain the sample entropy vector shown in (11).


(11) $$\begin{array}{l} En\left(M\right)=\left[En\left(S_{i}\right),\;En\left(S_{2}\right),\ldots ,En\left(S_{p}\right)\right] \end{array}$$

The above is the calculation progress of sample entropy, in which m is usually set as 2; r is usually set as 0.25 × std, where std refers to the standard deviation of the original time sequences (Nixon and Aguado, 2019). Next, the filtering process is performed. The collected tactile data may contain a certain amount of noise due to the instability of the finger motor and fluctuations in the grasping process, but the noise is small and discontinuous. For such noise, the median filtering is adopted for denoising. The basic formula of the median filtering is shown in (12).


(12) $$\begin{array}{l} F_{ji}=Med\left\{F_{\left(j-z\right)i},\ldots ,F_{\left(j-1\right)i},\;F_{ji},\;F_{\left(j+1\right)i},\ldots ,F_{\left(j+z\right)i}\right\};j\leq m,\\ z=\left\lceil \frac{T-1}{2}\right\rceil \end{array}$$

Where, *F*_*ji*_ represents the data collected by the i-th sensor for the j-th time, m is the number of a tactile sample, and is the ceil function, representing the smallest integer not less than this value. The noise distributions of the sensors are various for different samples or gripper positions. The higher the noise, the more complex the samples. In this paper, the filtering template is updated based on the sample entropy size, and the calculation formula of the update template size T (the initial value is set to 3) is shown in (13).


(13) $$\begin{array}{l} T_{i}^{En}=T_{i}\times En\left(S_{i}\right) \end{array}$$

The final denoised time sequences are shown in (14). The processing of the data filtering can more accurately reflect the object characteristics and improve the recognition accuracy.


(14) $$\begin{array}{l} S_{i}={\left[F_{1i},\;F_{2i},\ldots ,F_{mi}\right]}_{Med}^{T} \end{array}$$

## 3. The visual-tactile fusion network

In this section, we discuss our visual and tactile fusion framework in detail. While visual images are useful for representing global information such as object shape, size, and contour, touch provides local information related to properties such as softness, hardness, and material. To ensure that both global and local details are taken into consideration when performing feature expression, we propose a comprehensive visual-tactile fusion network model based on the adaptive dropout algorithm. This approach helps to preserve the structural information of visual images while effectively capturing fine-grained features with discrimination and adjusting the weight of visual and tactile information based on sample quality, leading to significantly enhanced object recognition accuracy.

### 3.1. The VTN model

**Figure 4:**
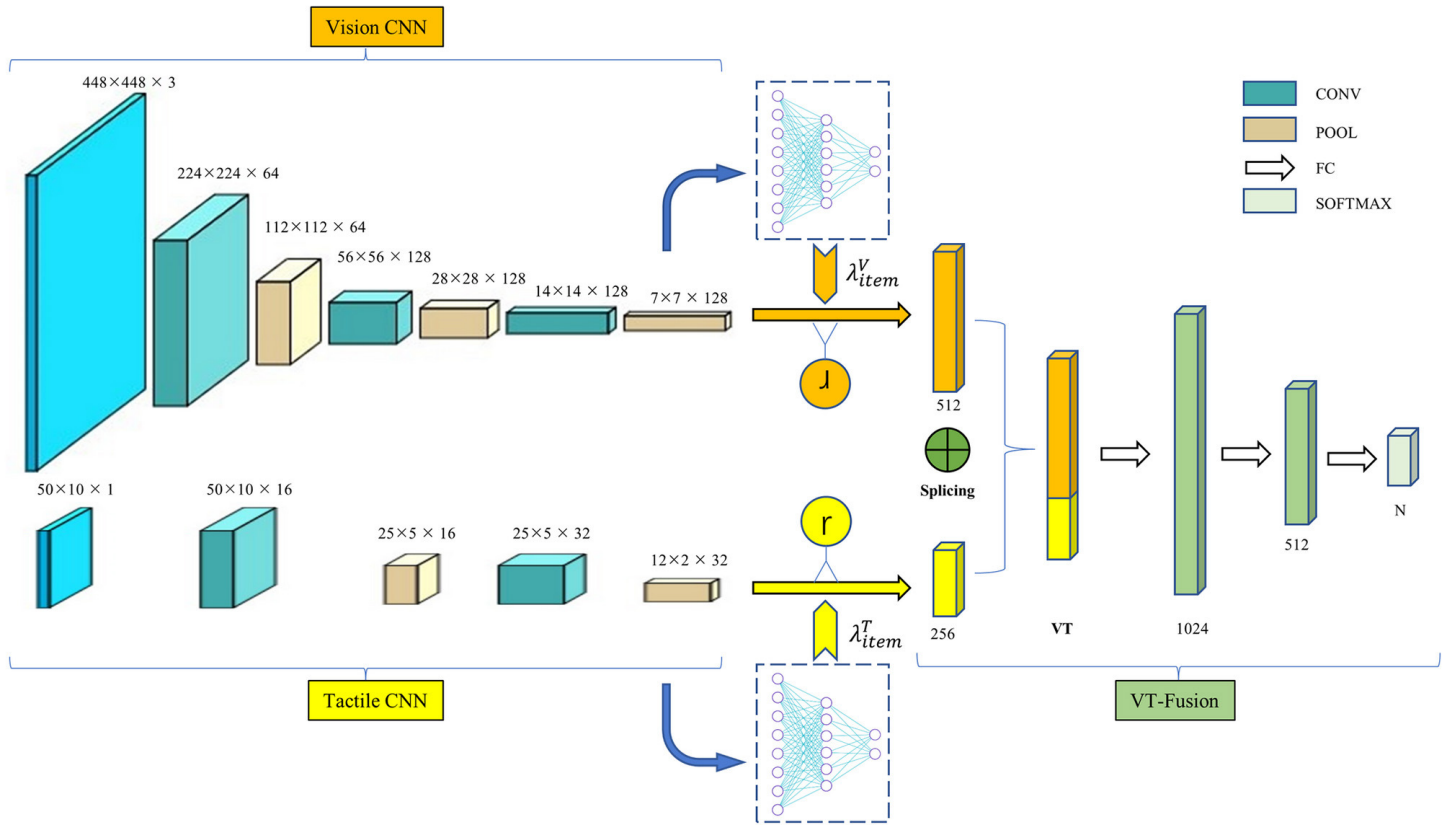
The framework of the VTN model. The model consists of a multi-branch CNN feature extraction module, a dropout module, and a feature fusion module. The input is the image acquired by the visual sensor and tactile sensor corresponding to the same object, and the output is the object attributes.

To achieve feature fusion, we propose a visual-tactile feature fusion network, as illustrated in Figure 4. This network combines visual and tactile feature vectors extracted from the input data to calculate and learn feature vectors. The visual input component is an image acquired using a camera, while the tactile input is generated by the multi-finger hand and the tactile sensor from Section 2. We design separate feature extraction networks for each source of information, which fuse at the final connection layer. The model comprises a multi-branch CNN feature extraction module, a dropout module, and a feature fusion module. It takes as input images obtained from both the visual and tactile sensors, which are processed through their respective CNN feature extraction modules. Since visual and tactile images differ significantly in size and composition, it is not possible to extract features from both sources of images simultaneously using the same network. To address this, branch CNN modules are used to design network structures specific to the different image features, producing two feature blocks: global features and fine-grained features containing local details. Once the visual-tactile fusion feature (VT) is obtained by splicing the visual and tactile feature blocks, it is processed through the fully connected module to produce the classification result.

The multi-branch CNN feature extraction module consists of two different sub-modules. The first sub-module is the visual feature network, which takes visual images collected by the camera as input. This sub-module has a network structure consisting of three convolutional layers and three pooling layers. The second sub-module is the tactile feature network, which has a simpler network structure due to the small size of the tactile image. It comprises two convolutional layers and two pooling layers, with network composition and parameters shown in the accompanying table. The output layer features of the two sub-modules are then concatenated to obtain visual-tactile fusion features, which are processed through the Softmax layer to produce the classification results.

The dropout layer is included as part of the fusion layer, which is described in the next section. A fusion algorithm is designed to combine visual features and tactile features in the fusion layer, resulting in the VT features. The fusion formula is shown in (15).


(15) $$VT\left(I_{v},\;I_{t},\;V,\;T\right)=\frac{V(I_{v})}{{\left\|V(I_{v})\right\|}_{2}}⊕\frac{T(I_{t})}{{\left\|T(I_{t})\right\|}_{2}}=\left(\begin{array}{l} v_{1}\\ v_{2}\\ \vdots \\ v_{m}\\ t_{1}\\ t_{2}\\ \vdots \\ t_{n} \end{array}\right)$$

Where *I*_*v*_ refers to the input visual image, and *I*_*t*_ refers to the input tactile image. *V* refers to the visual neural network mapping function, and the visual features are obtained by feature extraction from the visual image. *T* refers to the visual neural network mapping function, and the tactile features are obtained by feature extraction from the tactile image. ⊕ refers to the feature splicing operation, and the fused VT feature is obtained by splicing the visual and tactile features. The visual and tactile features need to be regularized separately before fusion.

### 3.2. Improved model based on the dropout optimization algorithm

**Figure 5:**
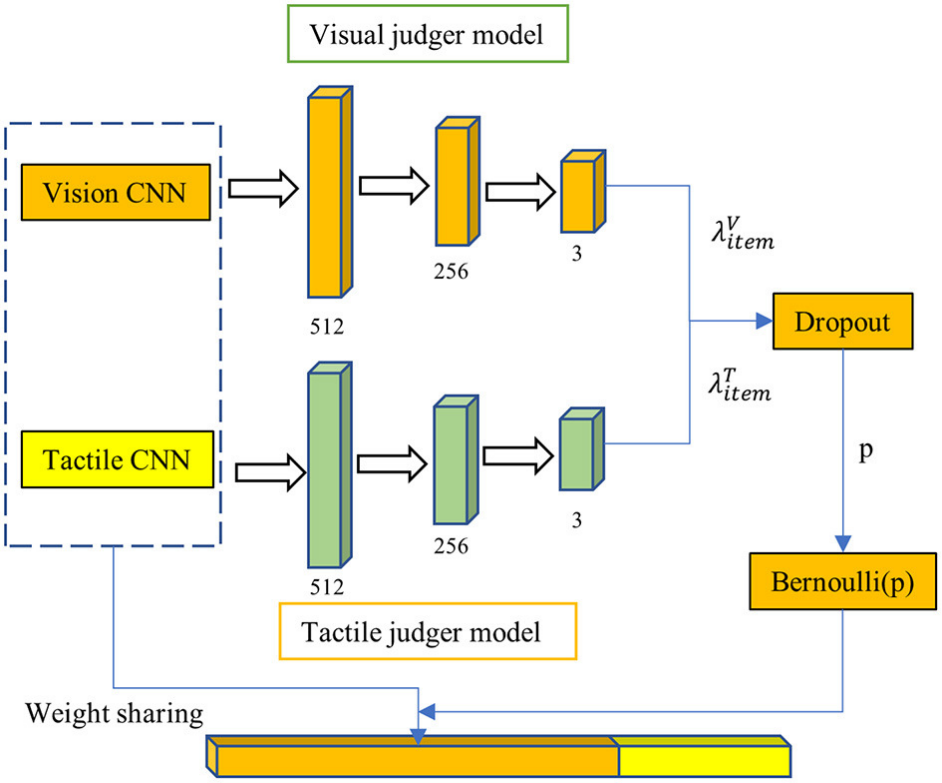
The dropout model. The visual image discrimination network and the tactile image discrimination network will be trained first before the training of the fusion network. This output represents the visual or tactile image's reliability in this recognition.

Under the influence of factors such as the quality of the collected data and the object material, visual data and tactile data reflect the true attributes of the object differently. For instance, visual samples can more effectively express the characteristics of objects with prominent colors, while tactile data is more representative of deformable objects. Additionally, visual data reliability decreases when objects are partially occluded, and tactile data is susceptible to noise when grasping is unstable. Therefore, it is essential to control the degree to which visual and tactile data impact the recognition results during the process of visual-tactile fusion. To achieve this, we propose an adaptive dropout model based on sample quality, illustrated in Figure 5. The adaptive dropout model consists of two image quality judger models, one for visual images and one for tactile images. Based on the quality scores assigned by these models, the dropout algorithm updates the dropout parameters of the fully connected layer prior to visual and tactile feature fusion. The better the image quality, the lower the dropout probability, and the greater the contribution of the image to recognition. The visual and tactile image discrimination networks are trained before the fusion network to enable discrimination of the output in the object recognition network, representing the reliability of visual and tactile inputs in recognition. This output is then directly applied to the fully connected layer before fusion as the dropout parameter to adjust the relative proportion of visual and tactile images in the recognition algorithm. This ensures that the negative impact of visual occlusion or tactile noise is avoided.

The dropout parameter p is determined by discriminating network output and neuron connection strength with the formula (16). Where, $\lambda_{item}^{V\left(T\right)}$ refers to the image quality score output by the visual discrimination network or the tactile discrimination network, and ℤ_*i*_(*t*) refers to the connection strength of a single neuron in the network, with the calculation formula shown in (17). *w*_*ij*_(*t*) refers to the weight between neurons *i* and *j* in the t-th iteration, and π_*ij*_(*t*) refers to the activation state of any neuron connected to neuron i in the network, with the value set as 0 or 1, 1 for active, and 0 for not active.


(16) $$\begin{array}{l} p=\left(1-\lambda_{item}^{V}\right)\cdot e^{-{\mathbb{Z}}_{i}\left(t\right)} \end{array}$$

(17) $$\begin{array}{l} {\mathbb{Z}}_{i}\left(t\right)=\frac{\underset{j\neq i}{\sum }w_{ij}\left(t\right)\pi_{ij}\left(t\right)}{\underset{i}{\sum }\underset{j\neq i}{\sum }w_{ij}\left(t\right)\pi_{ij}\left(t\right)} \end{array}$$

The Bernoulli method (Gal and Ghahramani, 2016) is utilized to randomly exclude some neurons in the hidden layer from the calculation with a probability of *p*. The dropout algorithm flow is depicted in Table 1. This approach has two significant advantages. Firstly, it makes model parameter training less dependent on the interaction of hidden nodes with fixed relationships, preventing certain features from only being effective in the presence of specific features. Secondly, as it acts on the fusion layer, it establishes a more stable connection between visual and tactile features. Moreover, deactivating some neurons reduces the mutual exclusion between visual and tactile data for the same object and can adjust the weight of both data types in recognition based on the inactivation ratio to enhance the recognition rate. The specific functioning mode of the dropout model is to adjust the probability of its activation state based on two factors: the connection strength of the neuron and the quality of the input image, enhancing the model's generalization capability.

**Table 1:** The flow of the adaptive dropout algorithm.

**Algorithm 1. The adaptive dropout algorithm**	
1	Input: Maxitem (number of training epochs), Layer (fusion layer)
2	Output: p (probability of dropout)
3	**Training:**
4	For *iter* ≤ *Maxitem* do:
5	Update $\lambda_{item}^{V}$ by the neural network
6	For *i* ≤ *Length*(*Layer*) do:
7	${\mathbb{Z}}_{i}\left(t\right)=\frac{\underset{j\neq i}{\sum }w_{ij}\left(t\right)\pi_{ij}\left(t\right)}{\underset{i}{\sum }\underset{j\neq i}{\sum }w_{ij}\left(t\right)\pi_{ij}\left(t\right)}$
8	$p=\left(1-\lambda_{item}^{V}\right)\cdot e^{\text{-}{\mathbb{Z}}_{i}\left(t\right)}$
9	$r_{j}^{\left(l\right)}\sim Bernoulli\left(p\right)$
10	ỹ^(*l*)^ = *r*^(*l*)^×*y*^(*l*)^
11	$z_{i}^{\left(l+1\right)}=w_{i}^{\left(l+1\right)}y+b_{i}^{\left(l+1\right)}$
12	$y_{i}^{\left(l+1\right)}=f\left(z_{i}^{\left(l+1\right)}\right)$
13	End for
14	End for
15	**Testing:**
16	ℤ_*i*_(*t*) = 1
17	Repeating steps 5–13

Where, $z_{i}^{\left(l+1\right)}$, $y_{i}^{\left(l+1\right)}$, $w_{i}^{\left(l+1\right)}$, and $b_{i}^{\left(l+1\right)}$ respectively represent the input vector, output vector, weight vector and bias vector of the i-th neuron in the *l*+1 hidden layer. *r*^(*l*)^ is the vector of neuron obeying Bernoulli distribution with probability p. *Bernoulli* is the Bernoulli distribution function. The activation function *f* is the Sigmoid function.

### 3.3. Training

The fused features map the (m+n)-dimensional vector to a C-dimensional vector through three fully connected layers. The C-dimensional vector outputs the probability of the corresponding category by the Softmax classification layer. The entire network is a complete end-to-end system with all parameters jointly trained by backpropagation, and our network is trained by standard backpropagation.

The Xavier (Kumar, 2017) initialization is adopted to initialize the network weights. We use SGD (Gao et al., 2016) with a constant learning rate of 0.01, the momentum of 0.9, and cross-entropy (De Boer et al., 2005) as the loss function. The model is implemented on the PyTorch platform and trained on the NVIDIA 2080Ti server. This paper sets the batch size to 8 and trains for 1,000 rounds.

The training process is as follows:

1. First, the visual sample evaluation network and the tactile sample evaluation network are trained.
2. The CNN network weights trained in step (1) are shared and the fusion network is trained on this basis.

## 4. Experiments

The experimental platform consists of a three-fingered dexterous hand, an array of tactile sensor, a Kinect2 camera, and a robot arm of the UR5 robot. The UR5 robot arm is placed on the left side of the table, on which we have assembled the three-fingered dexterous hand with 10 tactile sensors. The dexterous hand is JODELL's JQ3-5, and tactile sensors are the thick-film piezoresistive sensors. There is a Kinect2 camera on the right side of the table to capture the object images. The recognition procedure of the robot is as follows: Firstly, the visual sensor detects the grasping posture (Chu et al., 2018) of the object, then the robot moves to the grasping position and performs the grasping action to acquire tactile data. If the current grasping method fails to pick up the object, posture detection will be performed again until the object is smoothly grasped and stable tactile data can be obtained. The current posture detection has an average success rate of 92.3%, and the grasping success rate is 97.4%. We only collected tactile data after successful grasping for recognition testing.

### 4.1. Data set introduction

To construct the visual-tactile dataset, cylindrical, square, and spherical shapes were selected from common objects found in daily life. Each shape included three different levels of material: soft (H = −1), medium (H = 0), and hard (H = 1). There are 27 different types of objects. Extensive experiments were conducted to collect visual and tactile data on each object, with 50 images captured for each object under varying shooting conditions, including different occlusion areas. The tactile sensor sequences obtained during the stable grasping stage were chosen as the tactile data. Each object was recorded 50 times, and each tactile sequence was divided into 50 sample points. Then, 40 visual images and 40 tactile sequences were randomly selected from each object for training, with the remaining samples reserved for testing. All samples were obtained under successful grasping conditions. In addition to conventional category labels, the image qualities of visual and tactile samples were classified into three levels: good (Q = 1), medium (Q = 0), and poor (Q = −1). The tactile data was also divided into three levels based on time entropy: good (Q = 1), medium (Q = 0), and poor (Q = −1).

**Figure 6:**
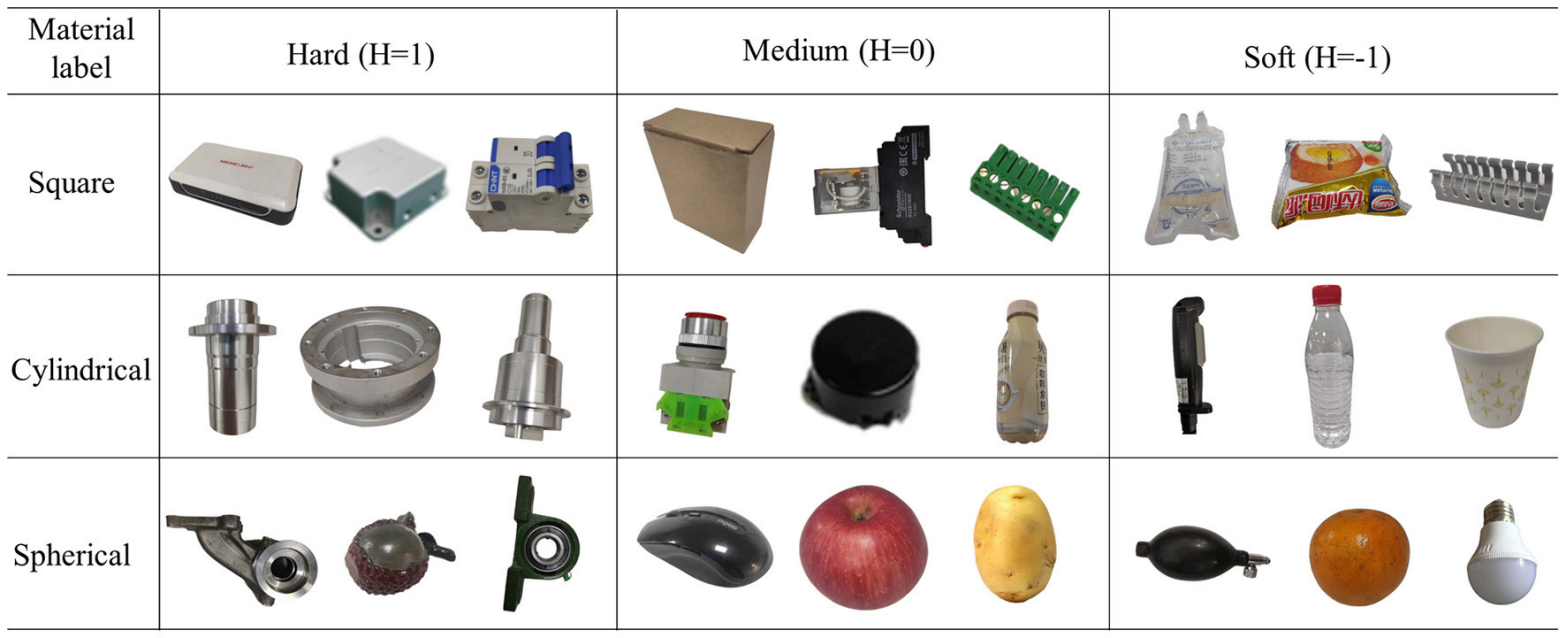
Example of the visual samples.

**Figure 7:**
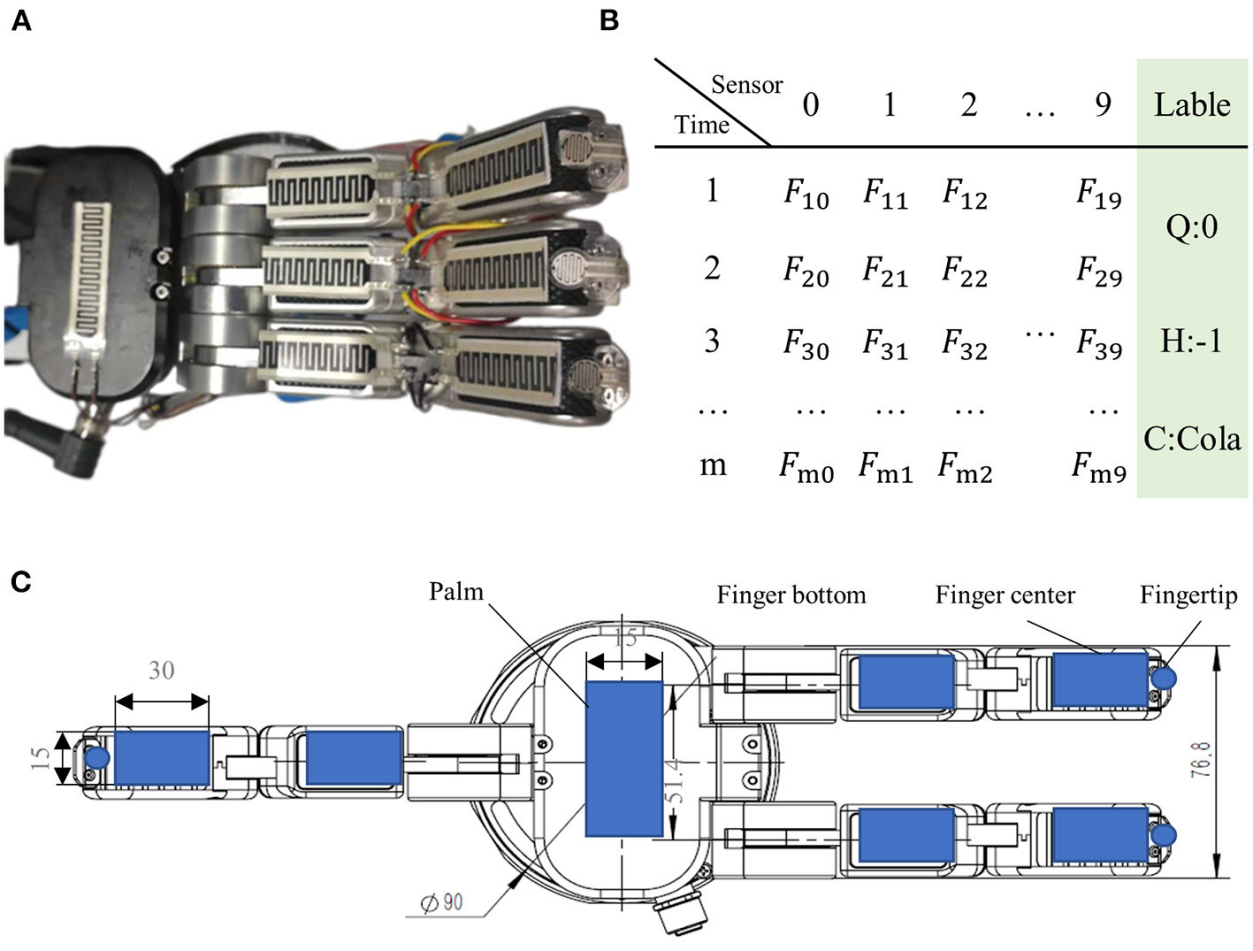
Examples of the tactile samples. **(A)** Magic hand, **(B)** tactile labels, which contain the tactile sensor feedback value *F* as well as the quality label (Q), material label (H), and category label (C), and **(C)** sensors distribution. There are 10 tactile sensors in the hand: the left fingertip, the middle fingertip, the right fingertip, the left finger center, the middle finger center, the right finger center, the left finger bottom, the middle finger bottom, the right finger bottom, and the palm.

Some visual and tactile samples are shown in Figures 6, 7. It is worth emphasizing that we include some components of the gyroscope in the sample to verify the algorithm's ability to recognize them.

### 4.2. Performance test experiments

#### 4.2.1. Performance comparison between tactile images and tactile sequences

The first challenge in achieving the fusion of visual and tactile data is the inconsistency between the modalities of these two types of data. To tackle this issue, this paper proposes a modality conversion method that converts tactile data into tactile images. To verify the feasibility of this approach, we compared the recognition results of an LSTM network that used the original tactile data and a CNN network that used the modality-converted tactile images. The CNN network comprises the tactile CNN part and the fully connected layer (excluding the VT layer) shown in Figure 4.

**Figure 8:**
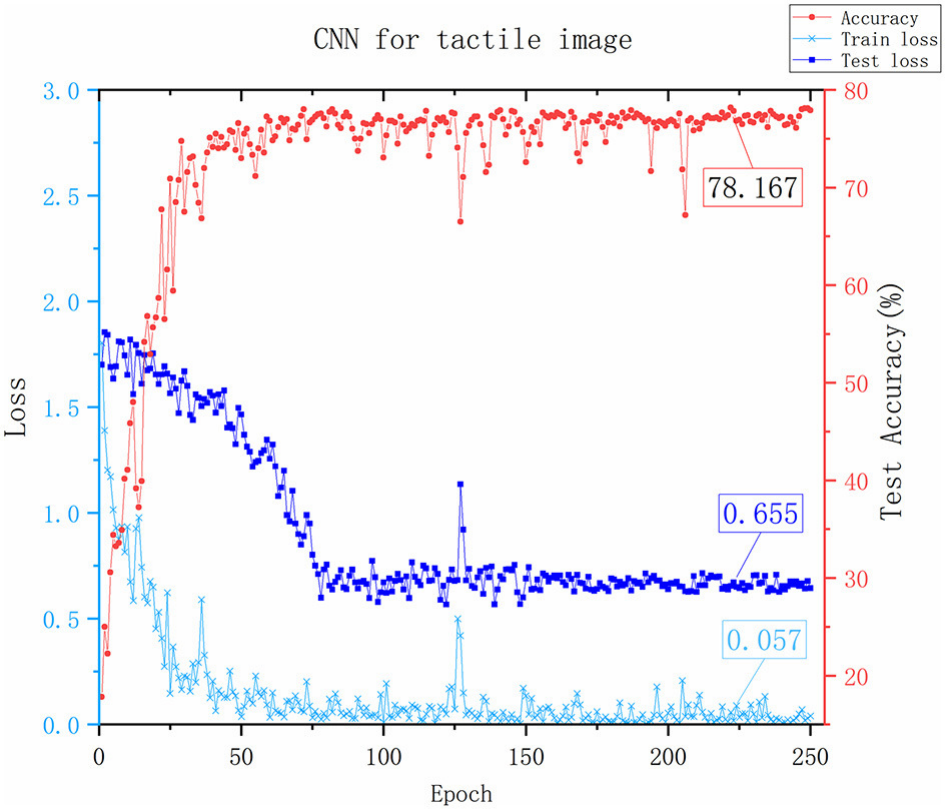
The results of the CNN Network. The x-axis represents the number of training rounds, the y-axis on the left represents the loss value, and the smaller the loss value, the better the network performance. The y-axis on the right represents the accuracy of recognition testing. The values marked in the figure is the last stable values.

**Figure 9:**
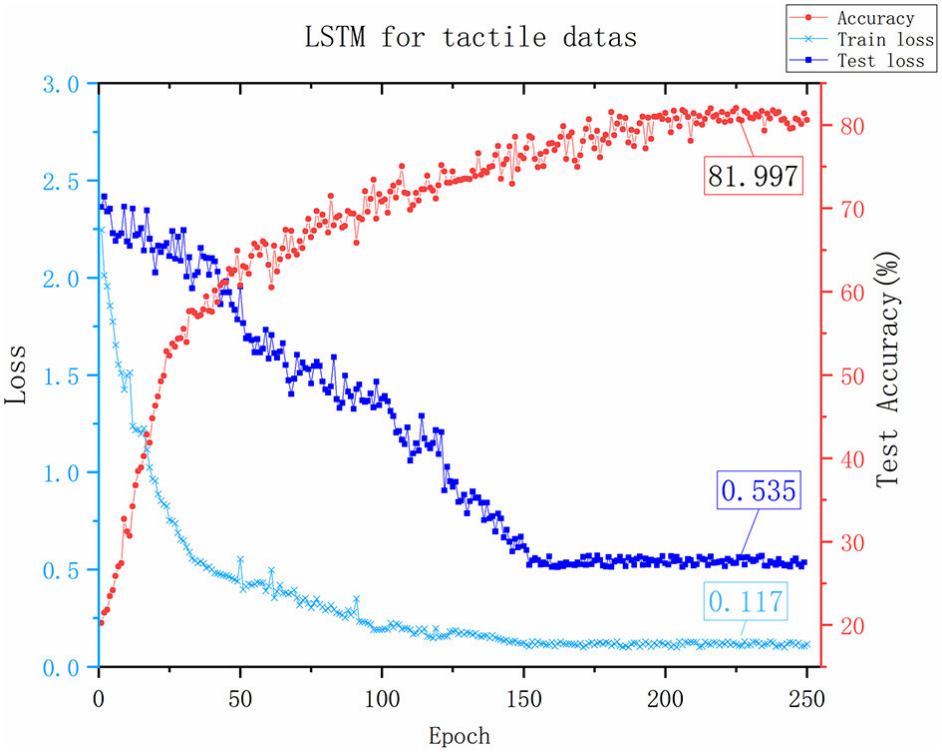
The results of the LSTM Network. The x-axis represents the number of training rounds, the y-axis on the left represents the loss value, and the smaller the loss value, the better the network performance. The y-axis on the right represents the accuracy of recognition testing. The values marked in the figure is the last stable values.

Although the network frameworks of the two groups of experiments were quite different, the purpose was not to verify network quality but to evaluate the effectiveness of tactile images. According to the experimental results in Figures 8, 9, the success rate of CNN recognition using tactile images reaches 78.167%, which was slightly lower than the 81.997% accuracy achieved by LSTM. This result was due to LSTM being a long short-term memory network and a time recurrent neural network that is suitable for processing and predicting time sequences. However, it was discovered from the change curve of the training loss and test loss that the CNN network can converge faster in the training process. The CNN network has a stronger feature extraction ability than the LSTM network, and once an effective feature is extracted, the positional relationship between it and other features is determined with strong stability. This group of experiments shows that converting tactile data into tactile images maintains data authenticity, and object attributes can be reflected.

**Figure 10:**
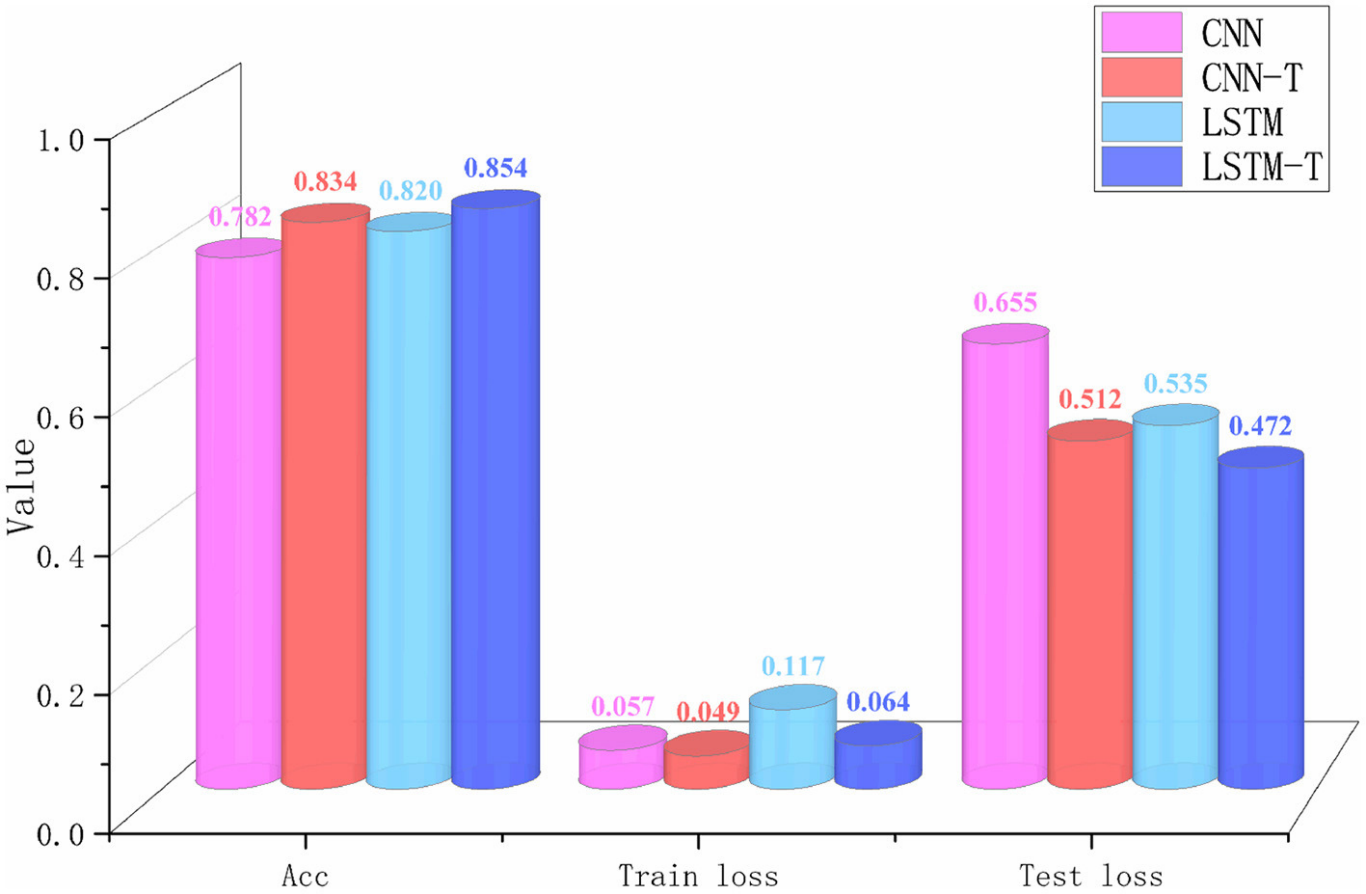
The results of different networks before and after filtering. There are four sets of experiments. The CNN-T and the LSTM-T indicate that the data is filtered before being input into the network.

It was also found that both networks fluctuated during training due to considerable interference in the tactile data acquisition process and noise in the data. To verify the denoising ability of the filtering algorithm proposed in this paper for tactile data, we conducted a comparative experiment on two different modality frameworks after filtering the original tactile data, as shown in Figure 10.

It is evident from the data in Figure 7 that the performance of the two different frameworks improved significantly after filtering, and the CNN network showed a better improvement than LSTM. Although the LSTM network is better at processing sequence data, the input sequences are too long, which significantly impacts the operational efficiency of LSTM. Therefore, LSTM network has a certain bottleneck that only extracts local information. However, CNN can efficiently run on longer inputs, obtain more stable hidden features in the sequences and enjoy more room for improvement. The construction of the tactile image and the CNN network of tactile image recognition provides a foundation for visual-tactile fusion.

#### 4.2.2. Comparative experiment of recognition effects of different models

Once the feasibility of the tactile modality conversion was verified, experiments on visual-tactile fusion were conducted to evaluate the performance of our visual-tactile fusion network. Three groups of comparative experiments were conducted, including visual recognition, tactile recognition, and visual-tactile fusion recognition. The visual network comprises the visual CNN part and the last three layers of the fully connected layer of the fusion network. Similarly, the tactile network refers to the tactile CNN part and the last three layers of the fully connected layer. Three indicators, including class accuracy, material property accuracy, and model size, were used to evaluate the performance of different algorithms. For example, the class of a ripe banana is “banana,” and the material property is “soft.” The data used in the three network models were all from our visual and tactile data sets. It was expected that the recognition algorithm could recognize more detailed material attributes of objects (and whether the material category is soft, medium, or hard) simultaneously as it recognizes the object category.

The experimental results in Table 2 show that the class accuracy and material category accuracy of the visual-tactile fusion perception are better than any single model recognition. The class accuracy of the visual-tactile fusion was as high as 99.3%, followed by visual class accuracy of 93.0%, and tactile class accuracy was the lowest. A comparison between the material property accuracy and class accuracy indicated that visual recognition was much better than tactile recognition in class accuracy. This was because visual images contain more object feature information, and visual recognition algorithms can identify most objects by extracting these features. However, visual material property accuracy declined with similar appearances but different materials. Tactile could address this issue. The tactile network's material property accuracy reached 95.2%, higher than the visual material property accuracy of 70.4%, demonstrating the difference between vision and touch. For rigid objects, the grasping force reaches its peak at the moment of grasping. For flexible objects, the grasping force increases gradually with the deformation of the object and reaches a peak eventually. Our visual-tactile fusion network integrates data from both modalities, enabling us to combine the advantages of both types of data and significantly improve the recognition effect. It was also found that tactile data had the advantage of simple information, few model parameters, and fast calculation speed, which led to no significant increase in model size after visual-tactile fusion compared to the visual network model.

**Table 2:** The recognition results of different models.

**Algorithms**	**Class accuracy**	**Material property accuracy**	**Model size (M)**
Tactile-only	0.826	0.952	5.3
Visual-only	0.930	0.704	26.5
Visual-Tactile fusion	0.993	0.978	29.8

**Figure 11:**
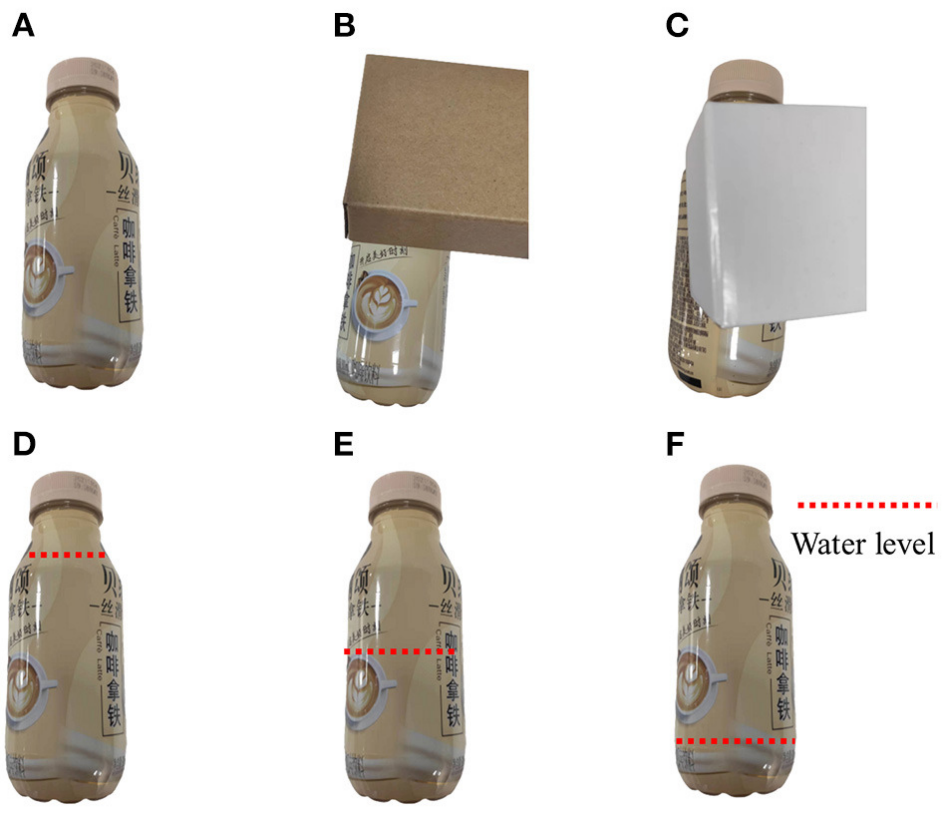
Examples of the water bottle samples. **(A)** No occlusion, **(B)** occlusion with 1/3, **(C)** occlusion with 2/3, **(D)** high level, **(E)** mediun level, **(F)** low level.

To further demonstrate the advantages of visual-tactile fusion, experiments were conducted to test the recognition ability of different algorithms in different object visibility scenarios by occluding water bottles. A total of 300 groups of experimental data were collected and tested, and the recognition rate of the object material attributes was evaluated with different algorithms using the samples shown in Figure 11. The water bottle attributes were divided into three levels of hardness (low, medium, and high) based on the water quantity in the bottle, and 80% of the collected data were used for training, with the remaining 20% used for testing. The test results are shown in Table 3.

**Table 3:** The recognition results of the water bottle.

**Algorithms**	**Class accuracy**			**Water quantity accuracy**
	**No occlusion**	**Occlusion with 1/3 of areas**	**Occlusion with 2/3 of areas**	
Tactile-only	0.833	0.850	0.833	0.967
Visual-only	0.967	0.867	0.617	0.483
Visual-Tactile fusion	1.000	0.933	0.883	0.950

The occlusion experiments showed that object occlusion had the most significant impact on the visual recognition algorithm, and the recognition accuracy of the visual algorithm dropped to 86.7 and 61.7%, respectively. It was challenging for the visual algorithms to extract complete and effective object features when the object was occluded, leading to recognition failures. However, the tactile algorithm only relied on the force feedback after contact between the finger and the object to identify the object, so occlusion did not affect the tactile algorithm's performance. Notably, occlusion had no noticeable impact on the visual-tactile fusion algorithm. There was no sharp drop in the accuracy of the visual-tactile fusion algorithm as observed with the visual algorithm. The visual-tactile fusion algorithm was equipped with a dropout mechanism, which allocated weights of the two types of data during fusion. When there was an occlusion in the visual image, the visual image quality discrimination network in the fusion algorithm could detect the image's low quality. The dropout probability of the fully connected layer of the visual part in the fusion layer was relatively high, which reduced the weight in the recognition, thus maintaining the recognition algorithm's accuracy at a high level.

In the water quantity attribute recognition experiment, the performance of the visual detection algorithm was found to be inadequate, with poor accuracy. However, the tactile detection algorithm demonstrated an impressive accuracy of 96.7%. Moreover, our visual-tactile fusion algorithm achieved an accuracy of 95.0%, which was not significantly affected by incomplete or unreliable visual data. These results demonstrate that our fusion algorithm is capable of effectively integrating visual and tactile features, leveraging the strengths of each modality and surpassing the performance of any standalone model. When faced with incomplete or unreliable feature information, the fusion algorithm adapts intelligently by adjusting the weights of the features and optimizing the classifier.

#### 4.2.3. Comparative experiment of model performance under different conditions

In order to validate the effectiveness of the proposed tactile image filtering algorithm and dropout algorithm in improving algorithm performance, we conducted four groups of comparative experiments by introducing each algorithm sequentially into the fusion algorithm with variable controls. The results of the tests are presented in Table 4.

**Table 4:** The results of model performance under different parameters.

**Variables**		**Class accuracy**	**Material property accuracy**
**Filtering**	**Dropout**		
×	×	0.959	0.915
✓	×	0.963	0.948
×	✓	0.985	0.956
✓	✓	0.993	0.978

From the experimental results, it is evident that both the tactile image filtering algorithm and the dropout algorithm positively impact the model's recognition performance. Comparison between the first and second groups of experiments shows that the filtering algorithm improves the model's material recognition performance. This is because the filtering algorithm enhances the quality of the tactile data, which is crucial for recognizing material properties that rely heavily on tactile features. In contrast, the experiments comparing the first and third groups demonstrate that the dropout algorithm significantly improves the model's overall performance, with a more pronounced improvement effect than the filtering algorithm.

Overall, the results of the comparative experiments confirm the effectiveness of the proposed visual-tactile fusion algorithm in robot object recognition tasks. Additionally, comparing the visual class accuracy rate of 93.0%, visual material property accuracy rate of 70.4%, and tactile class accuracy rate of 82.6% in Table 2, it is evident that even without optimization, the visual-tactile fusion network developed in this paper outperforms recognition by a single sensor. This further highlights the significant advantages of multi-information fusion over relying on single information at both the data and decision-making levels.

However, the material property accuracy rate of 91.5% is lower than the tactile accuracy rate of 94.8%, demonstrating the potential for two different information types to impact each other during the fusion process. The proposed dropout mechanism effectively reduces the negative impact of poor-quality samples on recognition results.

## 5. Conclusion

This paper proposes a visual-tactile fusion perception object recognition network VTN for robot recognition and grasping. The establishment of the filtering tactile images and the adaptive dropout algorithm provides a new optimization scheme for visual-tactile fusion perception tasks. Furthermore, it is demonstrated that tactile and visual signals are complementary, and combining data from two modalities can improve performance. The proposed model realizes the correlation between different modality information of vision and touch and provides an intelligent fusion mechanism for the fusion network. This paper has established data sets for training and testing with many recognition experiments, and the experimental results demonstrate the effectiveness and high accuracy of the proposed model. In the future, our model can be improved with more diverse data sets, and richer data will allow our network to identify more refined object attributes.

## Data availability statement

The original contributions presented in the study are included in the article/supplementary material, further inquiries can be directed to the corresponding author.

## Author contributions

ZD and GC conceived the study and put forward the methodology. ZD and ZW performed the data collection and pre-processing. GC and LS reviewed and edited the manuscript. All authors read and agreed to the published version of the manuscript.
